# Metabolic and Proteomic Profiles Reveal the Response of the ASD-Associated Resistant Strain 6-1 of *Lactobacillus plantarum* to Propionic Acid

**DOI:** 10.3390/ijerph192417020

**Published:** 2022-12-18

**Authors:** Rongrong Yu, Muchen Zhang, Temoor Ahmed, Zhifeng Wu, Luqiong Lv, Guoling Zhou, Bin Li

**Affiliations:** 1 College of Education, Zhejiang University of Technology, Hangzhou 310032, China; 2 Institute of Biotechnology, Zhejiang University, Hangzhou 310058, China; 3 Hangzhou Seventh People’s Hospital (HSPH), Hangzhou 310013, China

**Keywords:** ASD, propionic acid, metabolic profile, proteomic profile, resistance mechanism

## Abstract

Autism spectrum disorder (ASD) seriously affects children’s health. In our previous study, we isolated and identified a bacterium (*Lactobacillus plantarum* strain 6-1) that is resistant to propionic acid (PA), which has been reported to play a significant role in the formation of ASD. In order to elucidate the mechanism of the resistance to PA, this study investigated the change in the metabolic and proteomic profile of *L. plantarum* strain 6-1 in the presence and absence of PA. The results show that 967 and 1078 proteins were specifically identified in the absence and the presence of PA, respectively, while 616 proteins were found under both conditions. Gene ontology enrichment analysis of 130 differentially expressed proteins accumulated in the presence and absence of PA indicated that most of the proteins belong to biological processes, cellular components, and molecular functions. Pathway enrichment analysis showed a great reduction in the metabolic pathway-related proteins when this resistant bacterium was exposed to PA compared to the control. Furthermore, there was an obvious difference in protein–protein interaction networks in the presence and the absence of propionic acid. In addition, there was a change in the metabolic profile of *L. plantarum* strain 6-1 when this bacterium was exposed to PA compared to the control, while six peaks at 696.46, 1543.022, 1905.241, 2004.277, 2037.374, and 2069.348 *m*/*z* disappeared. Overall, the results could help us to understand the mechanism of the resistance of gut bacteria to PA, which will provide a new insight for us to use PA-resistant bacteria to prevent the development of ASD in children.

## 1. Introduction

Autism spectrum disorder (ASD) is a severe neurodevelopmental disorder which is characterized by abnormal social interactions, impaired language, and stereotypic and repetitive behaviors. In recent years, ASD has become a public health problem with a prevalence rate of 0.6–1.7% in children, while the number of children diagnosed with ASD is quickly increasing around the world [1,2,3,4,5]. In order to elucidate the cause of this disease, many studies have described and compared the composition of gut microbiota in children with and without ASD [6,7,8,9,10], which provided strong support for the change in the gut microbiome of ASD children compared to healthy controls. In general, these studies found that gut microbiota may play a fundamental role in the development and severity of ASD, which may open up the possibility for the effective therapy of these patients [11,12,13,14,15]. The meta-analyses showed that the relative abundance of gut bacteria in children with ASD and healthy controls could be grouped into 8 phyla and 17 genera by searching the main electronic databases as of February 2020 [16]. Three microbial markers were identified (*Faecalitalea*, *Caproiciproducens*, and *Collinsella*). There is no doubt that there was an altered microbial community structure of the gut microbiota in the ASD group compared to the healthy control group [17,18,19,20]. However, there has always been a lack of consistency in the changes in the gut microbiome across ASD studies. Indeed, there is an inconsistency in the types and abundances of bacteria present in the microbiota of individuals with ASD and controls [21,22,23,24,25].

In some cases, the production of bacterial metabolites such as indoles, lipopolysaccharides, and short-chain fatty acids was triggered by specific alterations to the gut microbiota, which were mostly observed in ASD patients [26,27]. Furthermore, there were significant differences between the ASD and healthy groups with regard to functional properties such as glycosyltransferase activity, galactose metabolism, and glutathione metabolism. Recent evidence indicates that specific metabolites can be used as diagnostic markers of ASD [28,29,30,31,32,33]. In particular, some metabolomic studies have shown that six metabolites, including inosine, methylguanidine, N-acetyl arginine, indoxyl sulfate, indole-3-acetic acid, and xanthurenic acid, could be considered as potential biomarkers [6,16,17]. The metabolic disruptions caused by altered gut microbiota may play an important role in the neurological pathophysiology of ASD by significantly increasing the number of bacterial species synthesizing branched-chain amino acids, but significantly reducing the number of probiotic species. In addition, the differential metabolites between ASD and TD groups were identified based on liquid chromatography–mass spectrometry (LC/MS), while some of them were found to be involved in the metabolic network of neurotransmitters such as GABA, dopamine, histidine, and serotonin.

On the other hand, the altered abundance of specific bacterial species may result in the differences in metabolites between the ASD and healthy groups [1,34]. Indeed, recent studies have reported the importance of propionic acid in developing ASD through different mechanisms [1,35,36]. For example, Guan et al. [35] showed that repeated intracerebroventricular infusions of propionic acid in adult rats produce behavioral and neuropathological changes, including hyperactivity, stereotypy, and repetitive movements, similar to those seen in ASD patients. Furthermore, Meeking et al. [36] found that mice that were injected with propionic acid display symptoms characteristic of ASD, such as social isolation and reduction in play behavior and oxidative stress.

Interestingly, our recent studies also revealed a difference in the number and composition of propionic acid-associated bacteria between ASD and healthy children, while *Lactobacillus plantarum* strain R6-1 isolated from ASD child fecal samples showed strong resistance to propionic acid at a high concentration [1]. Indeed, many enteric gut bacteria such as *B. vulgatus* normally secrete propionic acid, which can cross both the gut–blood and the blood–brain barrier [35,36]. Understanding the resistance mechanism will be beneficial for us to understand the role of propionic acid in the development of ASD. These findings suggest that a gut microbiome-associated therapeutic intervention may provide a novel therapeutic strategy for ASD by means of the modulation of propionic acid.

In order to elucidate the mechanism of the resistance to propionic acid, this study investigated and compared the metabolic and proteomic profile of *L. plantarum* strain 6-1 in the presence and absence of propionic acid based on the analysis of the LC-MS and proteome, respectively.

## 2. Materials and Methods

### 2.1. Bacterial Strain and Resistance to Propionic Acid

Strain 6-1 of *Lactobacillus plantarum* was obtained from our previous study, which was isolated from fecal samples from ASD children. The resistance of gut bacteria to propionic acid was evaluated by inoculating a single colony in nutrient broth with a 1.5 μg/mL concentration of propionic acid and then incubating the colony at 37 °C for 48 h. The resistant strain was routinely kept in nutrient broth [37] and was stored at −70 °C for further usage. Propionic acid was purchased from Shanghai Shenggong Bioengineering Technology Service Co., Ltd. (Shanghai, China).

### 2.2. Protein Extraction of Resistant Strain Exposed to Propionic Acid

Proteins were extracted according to the method of Shrivastava et al. [38]. In brief, bacterial colonies were taken from the NA medium plate, and then inoculated into 5 mL of NA broth. Following the overnight culture at 37 °C and 160 rpm/min, 2 mL of bacterial culture (approximately 10^8^ CFU/mL) was transferred to 200 mL of NB broth, and incubated in the same conditions until the OD600 was about 0.6. Following the centrifugation of the bacterial suspension at 6000× *g* for 10 min, cell pellets were lysed by adding total protein lysis buffer (SDT: 4% (*w*/*v*) SDS, 0.1M dithiothreitol (DTT), 100 mM Tris-HCl, pH 7.6) (the ratio of bacteria to SDT was 1:3), incubating at 95 °C for 3 min, sonicating for 2 min, and then centrifuging the mixture at 16,000× *g* for 5 min.

The supernatant was the initial protein extract, and the protein was quantified by Bradford analysis. Afterwards, the total protein was processed as described by Shrivastava et al. [38]. In short, the protein was first reduced with 10 mM DTT for 30 min at 37 °C, and then alkylated with 100 mM iodoacetamide (IAA) for 20 min at room temperature. Finally, the sample was digested with 0.05 M ammonium bicarbonate (ABC) and trypsin (the ratio of enzyme to protein was 1:50) and incubated overnight at 37 °C.

### 2.3. Peptide and Protein Identification

Peptides and proteins were identified as described by Cao et al. [39] based on the liquid chromatography–mass spectrometry (LC–MS/MS) analysis, which was carried out on an Orbitrap linear quadrupole ion trap (LTQ Orbitrap Elite) mass spectrometer (Thermo Fisher Scientific, Waltham, MA, USA) equipped with a nano-electrospray ion source. The sample linear concentration gradient separation conditions were as follows: the pre-column was a C18 reverse column (inner diameter 75 μm, length 2 cm, particle size 3 μm), the separation column was a C18 reverse column (inner diameter 50 μm, length 15 cm, particle size 2 μm), mobile phase A was water containing 0.1% formic acid, mobile phase B was acetonitrile containing 0.1% formic acid, the elution gradient was 3–90% acetonitrile (containing 0.1% formic acid) for 150 min, and the flow rate was 250 nL/min. The Orbitrap Elite was used for data detection, and Thermo Xcalibur Qual Browser was used for data collection.

### 2.4. Analysis of Proteins’ Differential Expression

The obtained mass spectrum data were identified by Proteome Discoverer 1.4, and the latest *L. plantarum* protein database (downloaded from http://www.uniprot.org/; accessed on 8 May 2022) was configured using SEQUEST HT to search the dataset. The peptides were extracted using high peptide confidence, and the false discovery rate (FDR) was <1%. After manual verification, the final list of proteins was prepared by identifying the proteins with the set threshold values, which were either a sufficient number of peptides or at least one peptide that was redundant enough to be considered reliable with acceptable scores. The redundant proteins and the redundant peptides from each protein were removed by verifying both the *m*/*z* value and the corresponding sequence. We adopted label-free proteomics to identify significantly different proteins (DEPs) for quantification. The protein requires at least one unique peptide. Under high-confidence conditions (PA/CK > 4 or PA/CK < 0.25), we identified up-regulated and down-regulated proteins. In addition, we displayed the proteins that distinguish the different clusters in a heatmap of the 130 differentially expressed proteins.

### 2.5. Protein Data Analysis

We used the UniProt database to perform functional annotations on the identified proteins, and performed functional enrichment of molecular functions, biological processes, and cellular components. The KEGG database (http://www.kegg.jp/kegg/pathway.html; accessed on 18 May 2022) was used to analyze the KEGG metabolic pathways of the identified proteins. We used the STRING database (https://string-db.org/; accessed on 22 May 2022) to analyze the interactions between proteins and visualized them in the Cytoscape 2.8 software (http://www.cytoscape.org/; accessed on 26 May 2022).

### 2.6. Lipopeptide Detection by MALDI-TOF-MS Analysis

Lipopeptides were analyzed for surfactin, iturin, and fengycin as previously described [40,41], using matrix-assisted laser desorption ionization–time of flight mass spectrometry (MALDI-TOF-MS). The propionic acid-treated and control strains were cultured in NA medium for 48 h, and the bacterial supernatant was collected for the detection of metabolites by recording mass spectra on a MALDI-TOF mass spectrometer (Bruker Daltonik GmbH, Leipzig, Germany) equipped with a 337 nm nitrogen laser for desorption and ionization.

## 3. Results and Discussion

### 3.1. Number of Identified Total Proteins

**Figure 1:**
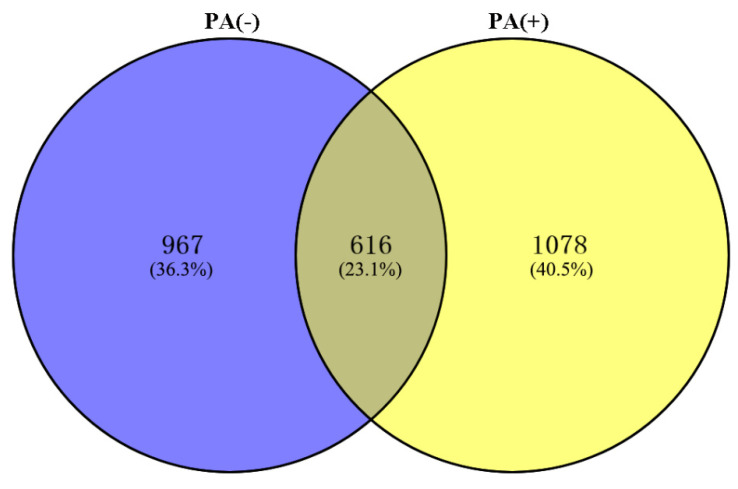
The number of total proteins identified in *Lactobacillus plantarum* strain 6-1 in the presence and absence of propionic acid.

Although it is well known that the gut bacteria are involved in ASD [42,43,44,45], the resistance mechanism of the gut bacteria to propionic acid is still unclear. In order to study the resistance of *L. plantarum* strain 6-1 to propionic acid as a whole at the protein level, the proteomes of strain 6-1 in the presence and absence of propionic acid were compared, and a total of 2661 proteins unique to strain 6-1 were identified. This result indicates that there was a difference in the number of proteins in the absence (Table S1) and presence (Table S2) of propionic acid, while a number of proteins were found under both conditions. In detail, 616 proteins are shared between the proteomes in the presence and absence of propionic acid, 967 proteins are unique to the absence of propionic acid, and 1078 proteins are unique to the presence of propionic acid (Figure 1).

Obviously, this result indicates that propionic acid greatly influences the expression of the proteome of *L. plantarum* strain 6-1. In agreement with the data in this study, previous studies have recorded significant changes in the protein profile of *L. plantarum* strain 423 that tolerates acidic pH when exposed to pH 2.5 by using a gel-free nano-LC–MS/MS proteomics approach [46]. Indeed, acid tolerance has been considered as an important characteristic of gut bacteria [47,48].

**Figure 2:**
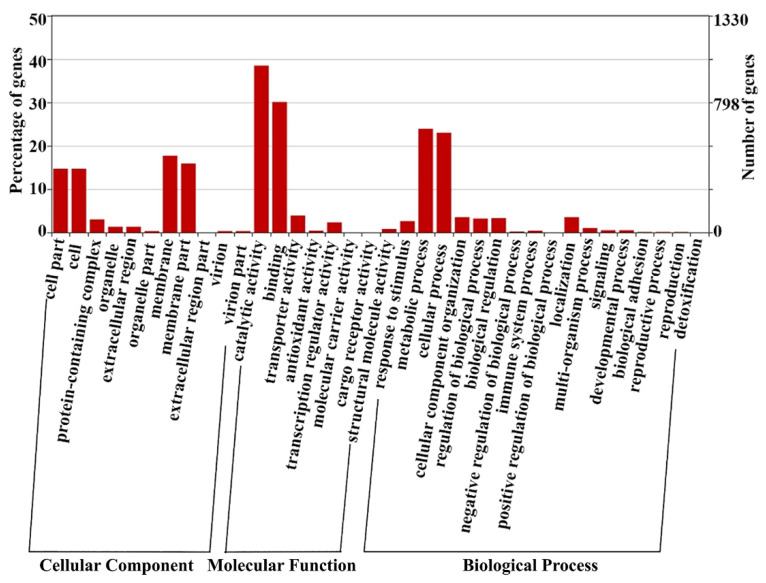
The number of proteins belonging to various functional categories identified in *Lactobacillus plantarum* strain 6-1 in both the presence and absence of propionic acid.

Most proteins contain at least two peptides identified by MS, and the remaining proteins are identified based on a single high-confidence peptide with 95% confidence. GO analysis results show that the identified proteins of *L. plantarum* strain 6-1 cover a wide range of cell components, molecular functions, and biological processes, mainly including the following functional categories: membrane (17.7%), membrane part (15.9%), catalytic activity (38.5%), binding (30.1%), metabolic process (23.9%), and cellular process (23%) (Figure 2).

### 3.2. Differentially Expressed Proteins

**Figure 3:**
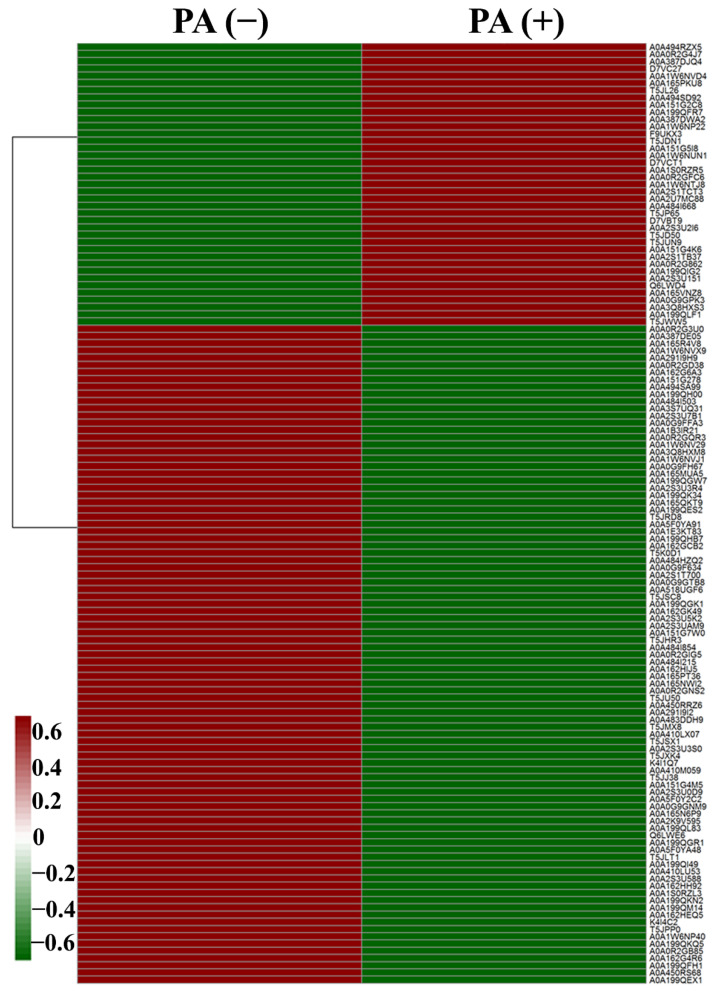
The heatmap showing the expression profiles of differentially expressed proteins (DEPs) of *Lactobacillus plantarum* strain 6-1 in the presence and absence of propionic acid.

The results of heatmap analysis show that 130 proteins (Table S3) in *L. plantarum* strain 6-1 showed significant differences in expression in the presence and absence of propionic acid. Obviously, propionic acid exhibited a great influence on the protein expression of *L. plantarum* strain 6-1. Among the proteins, 39 were up-regulated by propionic acid, while 91 proteins were down-regulated. These differentially expressed proteins may play an important role in bacterial resistance to the stress of propionic acid (Figure 3, Table 1).

In detail, propionic acid caused a more than 100-fold increase in the expression of DNA helicase, uncharacterized protein, and glutathione synthetase. In contrast, propionic acid resulted in a more than 100-fold reduction in the expression of flagellar protein FliS, N-acetylglucosaminyldiphosphoundecaprenol N-acetyl-beta-D-mannosaminyltransferase, coenzyme A biosynthesis bifunctional protein CoaBC, aminotransferase, serine tRNA ligase, and TetR family transcriptional regulators (TFRs).

The role of these differentially expressed proteins in environmental stresses has been documented in other bacteria. For example, the widely distributed TFRs in bacteria have been found to be able to regulate a wide range of physiological processes, from basic carbon metabolism and nitrogen metabolism to quorum sensing and antibiotic biosynthesis. Indeed, TFRs can control the expression of the tetracycline efflux pump in bacteria by binding DNAs and ligands. On the other hand, small molecule ligands can inhibit or promote TFRs to control target gene expression by inducing conformational changes in TFRs. 

In agreement with the results of this study, a previous study [46] also found that 97 proteins were detected as being more abundant, and 12 proteins were detected solely when *L. plantarum* strain 423 was exposed to pH 2.5. In acid-stressed cells, the utilization of a variety of carbohydrate sources in a glucose-rich environment, general stress response proteins, altered pyruvate metabolism, increased lysine biosynthesis, and a significant oxidative stress response were observed. The accumulation of basic compounds also seemed to play an integral role in bacterial response to acid stress. Furthermore, a marked decrease was also observed in proteins involved in transcription, translation, cell walls, cell division, and phospholipid biosynthesis. Functional analysis of the most abundant protein revealed that this protein was involved in survival during acid stress. 

### 3.3. Gene Ontology Enrichment Analysis

**Figure 4:**
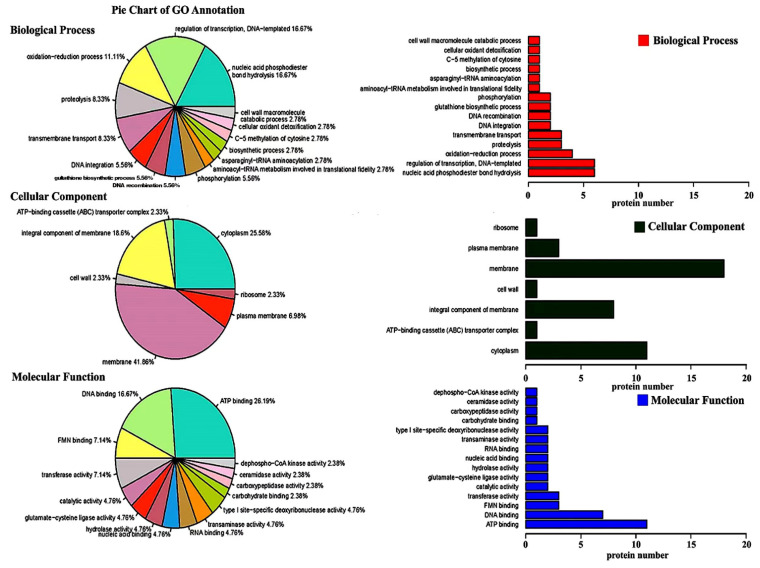
Gene ontology enrichment analysis of differentially expressed proteins of *Lactobacillus plantarum* strain 6-1 in the presence and absence of propionic acid.

GO annotation and GO enrichment of the identified proteins were carried out by using the UniProt database. The results showed that 121 out of the 130 differentially expressed proteins were categorized into three main categories, including biological processes, cellular components, and molecular function (Figure 4; Table S4). In detail, 36 proteins were speculated to be involved in the biological process, 43 proteins were speculated to be involved in the cellular component, while 42 proteins were speculated to be involved in the molecular function.

Among the 36 differentially expressed proteins involved in biological processes, two main categories of proteins were associated with nucleic acid phosphodiester bond hydrolysis and the regulation of DNA-templated transcription, which accounted for 16.67% (six) and 16.67% (six), respectively, of the total number of biological process proteins. These proteins have been reported to be involved in the unraveling of the DNA molecular structure and mRNA transcription, which may be used to selectively express proteins related to propionic acid stress in *L. plantarum* strain 6-1.

Among the 43 differentially expressed proteins involved in cellular components, two main categories of proteins were associated with the membrane and cytoplasm, accounting for 41.86% (18) and 25.58% (11), respectively, of the total number of cellular component proteins. It is well known that the cell membrane is the main target of environmental pressure in bacteria. This may explain the fact that a high ratio of membrane-related proteins in *L. plantarum* strain 6-1 were differentially expressed under propionic acid stress. In agreement with the results of this study, previous studies have also shown that the cell membrane can help bacteria maintain cell viability under acidic conditions in various ways [49].

Among the 42 differentially expressed proteins involved in molecular function, two main categories of proteins were associated with ATP binding and DNA binding, which accounted for 26.19% (11) and 16.67% (7), respectively, of the total number of molecular function proteins. It is well known that pH steady state is the regulation of the inside and outside pH of the cell, which is very important in affecting cell growth and metabolism, as well as protein and nucleic acid synthesis. pH homeostasis is the result of the interaction of multiple transport systems on the cell membrane. The transport of ions requires ATP to provide energy [34,50]. Therefore, it can be inferred that *L. plantarum* strain 6-1 may up-regulate the expression of transport system-related proteins when faced with propionic acid stress, such as A0A0R2G4J7 (ABC superfamily ATP binding cassette transporter) and A0A2S3U2I6 (calcium-transporting ATPase), so as to pump excess protons out of the body to maintain pH homeostasis by using the energy of ATP.

### 3.4. Pathway Enrichment Analysis

**Figure 5:**
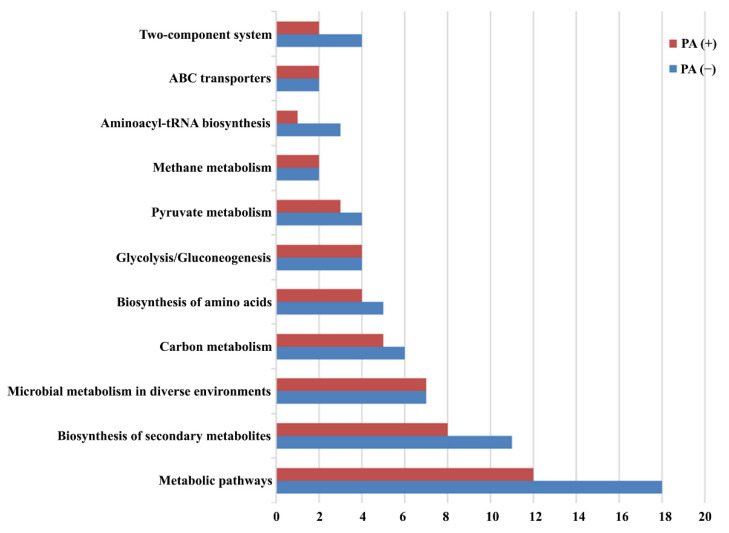
Pathway enrichment analysis of *Lactobacillus plantarum* strain 6-1 in the presence and absence of propionic acid.

The proteins identified in the absence (Table S5) and presence (Table S6) of propionic acid were further analyzed for KEGG metabolic pathways (Figure 5). The related proteins covered most of the metabolic pathways, including amino acid synthesis, carbohydrate metabolism, ABC transporter, and other pathways. It was found that *L. plantarum* strain 6-1 treated with propionic acid was affected in the metabolic pathways and biosynthesis of secondary metabolites. The treatment of propionic acid affected the metabolic pathways of *L. plantarum* strain 6-1 to some extent. In general, the number of proteins involved in various metabolic pathways of *L. plantarum* strain 6-1 was 50 and 66 in the absence and presence of propionic acid, respectively.

In detail, the number of proteins belonging to the two-component system, ABC transporters, aminoacyl-tRNA biosynthesis, methane metabolism, pyruvate metabolism, glycolysis/gluconeogenesis, biosynthesis of amino acids, carbon metabolism, microbial metabolism in diverse environments, biosynthesis of secondary metabolites, and metabolic pathways was 2, 2, 1, 2, 3, 4, 4, 5, 7, 8, and 12, respectively, in the presence of propionic acid, while the number of corresponding proteins was 4, 2, 3, 2, 4, 4, 5, 6, 7, 11, and 18, respectively, in the absence of propionic acid. This result suggests that propionic acid was able to affect various metabolic pathways of *L. plantarum* strain 6-1. Thus, it can be inferred that the missing proteins may be highly associated with propionic acid.

Obviously, these proteins associated with propionic acid are well known to be important for biological functions in bacteria. For example, two-component systems used as signaling pathways have been found to be able to regulate a variety of bacterial characteristics, such as virulence, pathogenicity, motility, symbiosis, nutrient uptake, secondary metabolite production, metabolic regulation, cell division, etc. In agreement with the results of this study, this system has been reported to regulate physiological processes in response to environmental pressures and enable adaptation to changing conditions. On the other hand, this system can be also used as a potential target for antimicrobial drug design. In recent years, significant advances have been made in the understanding of the role of two-component systems in bacterial adaptation to various ecological environments. 

### 3.5. Protein–Protein Interaction Networks

**Figure 6:**
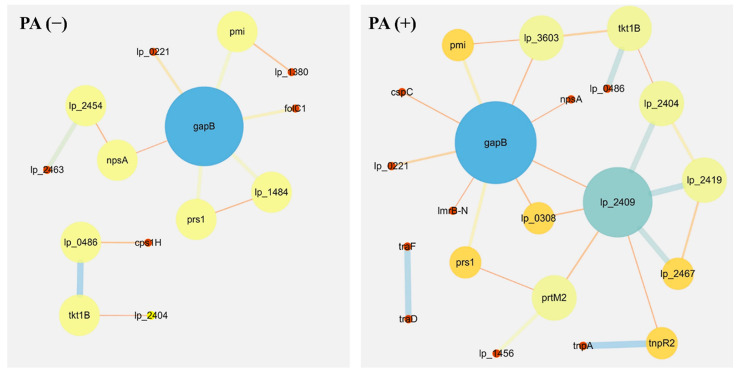
Protein–protein interaction networks of *Lactobacillus plantarum* strain 6-1 in the presence and absence of propionic acid.

In order to further analyze the proteomics data, we constructed a protein–protein interaction network. The main differentially expressed proteins from *Lactobacillus plantarum* strain 6-1 (Table 1) were involved in the protein–protein interaction network (Figure 6) in the absence (Table 2; Table S7) and presence (Table 3; Table S8) of propionic acid, respectively. Obviously, there was a difference in the composition of proteins in the absence and the presence of propionic acid. For example, lp_2463, lp_1484, folC1, cps1H, and lp_2454 were specific for the absence of propionic acid, lp_2419, lp_2467, lp_2409, prtM2, lp_3603, and cspC were specific for the presence of propionic acid, while tkt1B, gapB, and pmi lp_2404 were shared by the proteomes both in the absence and presence of propionic acid.

Roles in the metabolic and synthesis pathways have been observed for these differentially expressed proteins involved in the protein–protein interaction network from *Lactobacillus plantarum* strain 6-1 in the absence or presence of propionic acid. Furthermore, we found that the function of many proteins is highly related to prophages, regardless of the presence or absence of propionic acid. These results suggest that prophages may not only be highly associated with bacterial virulence, but are also involved in various biological functions of bacteria, such as bacterial survival, growth, and resistance to environmental stresses.

Interestingly, the results from this study show that the protein network of *L. plantarum* strain 6-1 in the presence of propionic acid is more complicated compared to that in the absence of propionic acid. For example, the protein of lp_2409 (prophage P2a protein 48 tape measure protein) only appears in the presence of propionic acid, and is connected with a series of newly added proteins, including prtM2 (foldase protein PrsA 2), lp_1456 (ABC transporter, permease protein), and tnpR2 (putative resolvase), which have been reported to be involved in the cell membrane, proton transport, and transcription and translation regulation. Therefore, it can be inferred that this interaction may be able to help strain 6-1 to deal with external propionic acid stress.

Our research has found that phage-related proteins may play an important role in the response of *L. plantarum* to the stresses of propionic acid. In agreement with the results of this study, Zhai et al. [51] reported that under cadmium stress, lp_2463 (prophage P2b protein 18, major capsid protein), lp_0641 (prophage P1 protein 18, DNA single-strand annealing protein RecT), lp_2444 (prophage P2a protein 13), and other phage-related proteins were significantly up-regulated in *L. plantarum*, which may help protect this bacterium against various external stresses.

### 3.6. MALDI–TOF Identification of Lipopeptides

MALDI-TOF mass spectra of *L. plantarum* strain 6-1 in the presence or absence of propionic acid are shown in Table 4, with a number of peaks in the mass range of 686.535 to 2648.81 *m*/*z*. Obviously, there was a difference in the peak area of the 28 peaks between the presence and absence of propionic acid. Among the shared 22 peaks, the addition of propionic acid caused the reduction in 21 peaks at varying degrees in the peak area, while only the peak at 1146.787 *m*/*z* was increased by propionic acid compared to the control. The peak at 1146.787 *m*/*z* has been assigned as iturins, which belong to a nonribosomal cyclic lipopeptide family of seven residues of α amino acids and one β amino acid. Previous studies have found that iturin is a kind of antibiotic produced by several strains of *Bacillus subtilis* with strong antimicrobial activity.

Interestingly, the six peaks at 696.46, 1543.022, 1905.241, 2004.277, 2037.374, and 2069.348 *m*/*z* disappeared in the presence of propionic acid. The cluster of peaks in the range of 1400–1600 *m*/*z* was assigned to fengycin isomers [52,53,54]. Therefore, the disappearance of the peaks at 1543.022 *m*/*z* revealed the absence of fengycin. Fengycin has been widely reported in Gram-positive and Gram-negative bacteria, which play an important role in the bacterial antagonism against other microbes. Therefore, it can be inferred that this resistant bacterium may be able to survive, but its antimicrobial ability can be reduced or even lost in the presence of propionic acid.

## 4. Conclusions

This study revealed the difference between the metabolic and proteomic profiles of *L. plantarum* strain 6-1 in the presence and absence of propionic acid, which was isolated and identified from fecal samples of ASD children in our previous study. Furthermore, an obvious difference was observed in protein–protein interaction networks in the presence and the absence of propionic acid. In addition, a great change was found in the metabolic profile of *L. plantarum* strain 6-1 when this bacterium was exposed to PA compared to the control, while six peaks disappeared. Therefore, it can be inferred that the resistance of *L. plantarum* strain 6-1 to propionic acid may be attributed to some specific lipopeptides and proteins, which are greatly increased or reduced by the presence of propionic acid. Overall, the results from study will help us to elucidate the mechanism of the resistance of gut bacteria to propionic acid, which will provide new insights for us to use this PA-resistant bacterium to prevent the development of ASD in children.

## Figures and Tables

**Table 1:** Differentially expressed proteins (>10- or <10-fold change) between the presence and absence of propionic acid.

UniProt Accession	Function Description	Fold Change
Down-regulation (>10-fold change)		
A0A0G9F634	Transposase	0.02
A0A0G9FH67	Aminotransferase	0.09
A0A2S1T700	Uncharacterized protein	0.03
A0A0R2G3U0	PTS family mannose fructose sorbose porter component IID	0.09
A0A2S3U5K2	ATP-dependent zinc metalloprotease FtsH	0.04
A0A2S3U7B1	Glutathione-disulfide reductase	0.07
A0A0R2GIG5	Flagellar protein FliS	0.01
A0A151G278	Integrase	0.10
A0A151G4M5	Uncharacterized protein	0.06
A0A3Q8HXM8	Uncharacterized protein	0.02
A0A162G4R6	DNA replication protein phage-associated	0.06
A0A162G6A3	Putative membrane protein	0.10
A0A162GCB2	Uncharacterized protein	0.06
A0A410LU53	Uncharacterized protein	0.08
A0A410LX07	Tape measure protein	0.07
A0A162HH92	Uncharacterized protein	0.02
A0A165MUA5	Type I restriction enzyme R protein	0.09
A0A484I215	N-acetylglucosaminyldiphosphoundecaprenol N-acetyl-beta-D-mannosaminyltransferase	0.00
A0A165NWI2	GMP reductase	0.05
A0A484I503	Uncharacterized protein	0.07
A0A484I854	Usp domain-containing protein	0.09
A0A165QKT9	Uncharacterized protein	0.06
A0A494SA99	Coenzyme A biosynthesis bifunctional protein CoaBC	0.01
A0A518UGF6	Restriction endonuclease subunit S	0.10
A0A199QEX1	Aminotransferase	0.01
A0A5F0Y2C2	POLAc domain-containing protein	0.05
A0A5F0YA48	Bifunctional glutamate-cysteine ligase GshA/glutathione synthetase GshB	0.05
A0A199QGR1	Transposase	0.06
A0A199QH00	UDP-glucose 6-dehydrogenase	0.05
K4I4C2	Tail tape measure	0.10
T5JJ38	TetR family transcriptional regulator	0.01
A0A1B3IR21	Relaxase superfamily protein	0.05
A0A1S0RZL3	Uncharacterized protein	0.03
T5JSC8	Oligopeptidase PepB	0.02
T5JU50	Serine tRNA ligase	0.01
A0A1W6NVX9	Conjugal transfer protein	0.05
T5K0D1	Asparagine tRNA ligase	0.07
Up-regulation (>10 fold change)		
A0A1W6NVD4	Uncharacterized protein	11.55
T5JP65	HTH lacI-type domain-containing protein	17.40
T5JUN9	2,5-diketo-D-gluconic acid reductase	24.93
T5JWW5	Chromosome partition protein Smc	18.05
T5JL26	5-formyltetrahydrofolate cyclo-ligase	24.40
T5JD50	Glutathione synthetase	210.94
A0A484I668	Uncharacterized protein	11.40
A0A199QFR7	Protein kinase	72.45
D7VBT9	Site-specific recombinase, phage integrase family	11.89
D7VC27	Glycosyltransferase, group 1 family protein	10.29
Q6LWD4	Putative transposase	17.71
A0A387DJQ4	Terminase small subunit	24.72
A0A151G5I8	Uncharacterized protein	119.47
A0A165PKU8	Functional role page for anaerobic nitric oxidereductase transcription regulator NorR	10.90
A0A2S3U2I6	Calcium-transporting ATPase	18.16
A0A2U7MC88	RpoD (fragment)	18.86
A0A2S3U151	DNA helicase	106.37

Fold change: propionic acid (+)/(−).

**Table 2:** List of the main DEPs from *Lactobacillus plantarum* strain 6-1 in the absence of propionic acid involved in protein–protein interaction (PPI) network.

Node	External_id	Function	Role in Metabolic and Synthesis Pathway
tkt1B	220668.lp_0491	transketolase, pyrimidine-binding domain	+
lp_2463	220668.lp_2463	prophage P2b protein 18, major capsid protein	+
lp_1484	220668.lp_1484	hypothetical protein	+
gapB	220668.lp_0789	glyceraldehyde 3-phosphate dehydrogenase	+
pmi	220668.lp_2384	mannose-6-phosphate isomerase	+
folC1	220668.lp_2321	formylTHF-polyglutamate synthase/folyl-polyglutamate synthase/hydrofolate synthase	+
cps1H	220668.lp_1184	glycosyltransferase (rhamnosyltransferase), family 2 (GT2)	+
lp_2454	220668.lp_2454	prophage P2a protein 3; DNA adenine methylase	+
lp_2404	220668.lp_2404	prophage P2a protein 53	+

**Table 3:** List of the main DEPs from *Lactobacillus plantarum* strain 6-1 in the presence of propionic acid involved in protein–protein interaction (PPI) network.

Node	External_id	Function	Role in Metabolic and Synthesis Pathway
lp_2419	220668.lp_2419	prophage P2a protein 38; minor head protein	+
tkt1B	220668.lp_0491	transketolase, pyrimidine-binding domain	+
lp_2467	220668.lp_2467	prophage P2b protein 14, terminase small subunit	+
lp_2409	220668.lp_2409	prophage P2a protein 48; tape measure protein	+
prtM2	220668.lp_3193	peptidyl-prolyl isomerase	+
gapB	220668.lp_0789	glyceraldehyde 3-phosphate dehydrogenase	+
pmi	220668.lp_2384	mannose-6-phosphate isomerase	+
lp_3603	220668.lp_3603	sugar-phosphate aldolase	+
cspC	220668.lp_0997	cold shock protein CspC	+
lp_2404	220668.lp_2404	prophage P2a protein 53	+

**Table 4:** Lipopeptides produced by *Lactobacillus plantarum* strain 6-1 in the absence and presence of propionic acid based on MALDI-TOF-MS mass spectra of whole cells.

*m*/*z*	Peak Area			*m*/*z*	Peak Area		
	PA (+)	PA (−)	PA (+)/(−)		PA (+)	PA (−)	PA (+)/(−)
686.535	9.86	11.9	0.83	2037.374	—	42.4	—
696.46	—	9.35	—	2059.347	101	194	0.52
911.601	21.4	26.6	0.80	2069.348	—	31.2	—
984.637	12.3	12.9	0.95	2091.317	64	126	0.51
1105.699	16.8	22.7	0.74	2093.502	17.7	32.9	0.54
1110.711	15.6	21.9	0.71	2108.472	40.2	57.9	0.69
1122.732	75.2	83.4	0.90	2168.432	36.1	82.7	0.44
1144.706	15.3	23.1	0.66	2190.411	220	439	0.50
1146.787	77.1	71.8	1.07	2205.39	25.2	27.3	0.92
1322.883	15	17.8	0.84	2207.556	312	649	0.48
1543.022	—	19.5	—	2223.534	25.5	49.6	0.51
1905.241	—	23.9	—	2533.751	51.7	84.9	0.61
1960.269	21.6	44.9	0.48	2632.834	467	1314	0.36
2004.277	—	22.1	—	2648.81	38.7	80	0.48

## Data Availability

Not applicable.

## Supplementary Materials

The following supporting information can be downloaded at: https://www.mdpi.com/article/10.3390/ijerph192417020/s1, Table S1. The identified total proteins of *Lactobacillus plantarum* strain 6-1 in the absence of propionic acid. Table S2. The identified total proteins of *Lactobacillus plantarum* strain 6-1 in the presence of propionic acid. Table S3. Significant differentially expressed proteins (DEPs) of *Lactobacillus plantarum* strain 6-1 in the presence of propionic acid. Table S4. Gene Ontology (GO) enrichment analyses of differentially expressed proteins (DEPs). Table S5. List of proteins from *Lactobacillus plantarum* strain 6-1 in the absence of propionic acid showing role in different metabolic and synthesis pathways. Table S6. List of proteins from *Lactobacillus plantarum* strain 6-1 in the presence of propionic acid showing role in different metabolic and synthesis pathways. Table S7. List of DEPs from *Lactobacillus plantarum* strain 6-1 in the absence of propionic acid involved in protein–protein interaction (PPI) network. Table S8. List of DEPs from *Lactobacillus plantarum* strain 6-1 in the presence of propionic acid involved in protein–protein interaction (PPI) network.
